# Chronic exposure to diesel particles worsened emphysema and increased M2-like phenotype macrophages in a PPE-induced model

**DOI:** 10.1371/journal.pone.0228393

**Published:** 2020-01-31

**Authors:** Alyne Riani Moreira, Thamyres Barros Pereira de Castro, Júlia Benini Kohler, Juliana Tiyaki Ito, Larissa Emídio de França Silva, Juliana Dias Lourenço, Rafael Ribeiro Almeida, Fernanda Roncon Santana, Jose Mara Brito, Dolores Helena Rodriguez Ferreira Rivero, Maria Isabel Cardoso Alonso Vale, Carla Máximo Prado, Niels Olsen Saraiva Câmara, Paulo Hilário Nascimento Saldiva, Clarice Rosa Olivo, Fernanda Degobbi Tenorio Quirino dos Santos Lopes

**Affiliations:** 1 Department of Clinical Medicine (LIM 20), School of Medicine, University of Sao Paulo, Sao Paulo, Brazil; 2 Institute of Medical Assistance to the State Public Servant (IAMSPE), Sao Paulo, Brazil; 3 University City of Sao Paulo (UNICID), Sao Paulo, Brazil; 4 Department of Immunology, Institute of Biomedical Sciences, University of Sao Paulo, Sao Paulo, Brazil; 5 Heart Institute (InCor) School of Medicine, University of Sao Paulo, Sao Paulo, Brazil; 6 Department of Bioscience, Federal University of Sao Paulo, Diadema, Sao Paulo, Brazil; 7 Department of Pathology (LIM 5), School of Medicine, University of Sao Paulo, Sao Paulo, Brazil; 8 Department of Bioscience, Federal University of Sao Paulo, Santos, Sao Paulo, Brazil; 9 Department of Clinical Medicine (LIM 16), School of Medicine, University of Sao Paulo, Sao Paulo, Brazil; 10 Department of Medicine, Nephrology Division, Federal University of Sao Paulo, Sao Paulo, Brazil; Telethon Institute for Child Health Research, AUSTRALIA

## Abstract

Chronic exposure to ambient levels of air pollution induces respiratory illness exacerbation by increasing inflammatory responses and apoptotic cells in pulmonary tissues. The ineffective phagocytosis of these apoptotic cells (efferocytosis) by macrophages has been considered an important factor in these pathological mechanisms. Depending on microenvironmental stimuli, macrophages can assume different phenotypes with different functional actions. M1 macrophages are recognized by their proinflammatory activity, whereas M2 macrophages play pivotal roles in responding to microorganisms and in efferocytosis to avoid the progression of inflammatory conditions. To verify how exposure to air pollutants interferes with macrophage polarization in emphysema development, we evaluated the different macrophage phenotypes in a PPE- induced model with the exposure to diesel exhaust particles. C57BL/6 mice received intranasal instillation of porcine pancreatic elastase (PPE) to induce emphysema, and the control groups received saline. Both groups were exposed to diesel exhaust particles or filtered air for 60 days according to the groups. We observed that both the diesel and PPE groups had an increase in alveolar enlargement, collagen and elastic fibers in the parenchyma and the number of macrophages, lymphocytes and epithelial cells in BAL, and these responses were exacerbated in animals that received PPE instillation prior to exposure to diesel exhaust particles. The same response pattern was found inCaspase-3 positive cell analysis, attesting to an increase in cell apoptosis, which is in agreement with the increase in M2 phenotype markers, measured by RT-PCR and flow cytometry analysis. We did not verify differences among the groups for the M1 phenotype. In conclusion, our results showed that both chronic exposure to diesel exhaust particles and PPE instillation induced inflammatory conditions, cell apoptosis and emphysema development, as well as an increase in M2 phenotype macrophages, and the combination of these two factors exacerbated these responses. The predominance of the M2-like phenotype likely occurred due to the increased demand for efferocytosis. However, M2 macrophage activity was ineffective, resulting in emphysema development and worsening of symptoms.

## Introduction

There is a strong association between exposure to air pollutants and an increase in hospital admissions for respiratory and cardiac diseases [1]. These deleterious effects, especially in the respiratory tract, are mainly attributed to particulate matter (PM) air pollutants less than 10 μm (PM10) or 2.5 μm (PM2.5) in aerodynamic diameter [1]. While the mechanisms underlying the adverse effects of PM on the respiratory and cardiac systems are not completely understood, the leading hypotheses emphasize inflammatory responses in the lung and the release of cytokines with local and systemic consequences [2–4]. Particulate matter in the lungs induce inflammation, exacerbate underlying lung diseases and reduce the efficacy of lung-defense mechanisms [2].

Considering all lung diseases, chronic obstructive pulmonary disease (COPD) is the most highly correlated with air pollutant exposure and increased global urbanization [5]. COPD is characterized by a persistent inflammatory response in the lungs to exogenous agents, and patients with this condition are more susceptible to the adverse effects of PM [6].

We previously showed that chronic exposure to urban levels of traffic-related PM10 worsens protease-induced emphysema in mice and is associated with an increase in macrophages, alveolar enlargement, parenchymal remodeling and oxidative stress [7].

Macrophages are the first line in recognizing tissue damage in response to PM and microorganisms [8]. These cells act to phagocytose and process particles when they cannot be cleared by mucociliary action [9,10]. Upon PM contact, macrophages are activated and produce proinflammatory cytokines, perpetuating the preexisting inflammatory process in lung diseases [11].

Depending on microenvironmental stimuli, macrophages can assume different phenotypes with different functional actions. In the presence of interferon-gamma (IFN-γ), macrophages can be polarized into the M1 phenotype, which is recognized by the release of the cytokine interleukin (IL)-12 and the chemokines CXCL-9 and CXCL-10, which possess proinflammatory, microbicidal and tumoricidal properties [12].

In contrast, in the presence of tumor necrosis factor-alpha (TNF-α), IL-4, IL-13 and IL-10, alternative activation persists, inducing the M2 phenotype. M2-like macrophages are responsible for the release of transcription factors such as interferon regulatory factor (IRF)-4 and the gene expression of arginase (Arg)-1, which is found in inflammatory zone (FIZZ) and molecule like chitinase type 1 (YM-1). These molecules are recognized for exerting anti-inflammatory and healing actions [13].

Additionally, M2-like macrophages are recognized by their capability to phagocytize apoptotic cells (efferocytosis) that are not efficiently eliminated by the mucociliary system [14–16]. The removal of apoptotic cells is essential in preventing inflammation progression [17].

It has been shown that fine particles can easily the distal areas of the lungs, a situation that impairs their removal and perpetuates their noxious effects [8,11]. As a result, the inflammatory response is maintained [9,10,18], and there is a consequent increase in the demand for phagocytosis of apoptotic cells [19].

Considering that fine particulate matter can reach the most distal parts of the lungs and the importance of macrophages in emphysema development, we aimed to verify the effect of exposure to diesel exhaust particles in an induced-emphysema model. We focused on macrophage phenotypes and the microenvironmental stimuli that could interfere with their polarization.

## Methods

The present study was approved by the Ethics Committee on Human and Animal Research of the School of Medicine of the University of São Paulo (protocol number 107/15). Male C57BL/6 mice, 6–8 weeks old, were used. All animals received humanitarian care in accordance with the National Institutes of Health Guide for the Care and Use of Laboratory Animals (NIH Publication No. 85–23, revised 1996).

### Induction of emphysema: Elastase-induced model

The animals were anesthetized with a combination of xylazine and ketamine (i.m., 5 mg/kg and 40 mg/kg, respectively) and then challenged with an intranasal instillation of 50 μL of PPE (Type I/ E1250; Sigma-Aldrich, St. Louis, MO, USA) at a dose of 0.667 IU.

### Exposure to pollutants

Animals were exposed in a whole body chamber to diesel exhaust particles for one hour, five times a week, for 60 days by a stationary diesel electrical generator (BD-2500 CFE; Branco, China) fitted via a simple connection on its exhaust tube that was divided into three compartments: an exhibition room, machine room and room in which the particles were generated. The exhibition room consisted of a bench with exposure chambers, a pressure-regulating valve and an apparatus that measured PM2.5, NOx, NO_2_ and NO levels [20,21].

The machine room contained the operating equipment (e.g., a HEPA filter, a compressed air system, pumping system and individual dosing of gases, electrical panels, and the condensing unit of the air cooling system). The room in which the particles and gases were generated, contained the electrical generator of the particles and gases (BD-2500, Branco, China) created from burning diesel. This system was connected to a bag for storage and hoses and exhaust ducts. The storage bag contained an entrance for atmospheric air. The vacuum pump captured the external air and directed it into the HEPA air filtration system (model number: VSWPSA 102–90 / 2P-BLR-12M-12M-6F-PLN; Purafil^®^, St. Louis, MO, USA) at a flow rate of approximately 150 liters per minute (L/min). The gases and particles were conducted to the inhalation chamber, which contained the animals.

The concentrations of PM2.5 (DataRam4^™^, Thermo Fisher Scientific Inc., Franklin, MA, USA), nitric oxide (NO) and nitrogen dioxide (NO_2_), along with temperature and relative humidity, were monitored in real time. After the gases passed through the chamber, they were directed out of the apparatus via a fan (see supplementary material). With this material already diluted in the bag, the gases and particles were directed to the inhalation chamber, where the mice were accommodated in separate transport boxes in groups to expose them by inhalation to the material from the burning of the fuel under study.

#### Exposure delineation

The mean concentration of PM2.5 within the chamber was adjusted to 577 μm/m^3^ to simulate the concentration that the mice would experience during 24 hours of exposure. This concentration is equivalent to a daily average of 24 μm/m^3^, which is below the daily average of PM2.5 recommended by the World Health Organization (25 μm/m^3^) [22].

At the concentration of PM2.5 inside the chamber, the mice received an average of 600 μg/m^3^ for 1 hour to simulate a concentration that would be received on 24 hours. In this context, these concentrations are equivalent to a daily average of 25 μg/m^3^, which is the maximal recommended daily concentration by the WHO (WHO Air quality guidelines for particulate matter, ozone, nitrogen dioxide and sulfur dioxide: Global update 2005) in their guidelines [22]. Our study aimed to represent a “real world” exposure in an urban scenario such as Sao Paulo, a city with approximately 6,065,157 cars, 421,630 diesel vehicles and 899,947 motorcycles that circulate daily on the main streets. In the last year, the maximal daily concentrations of PM2.5 in Sao Paulo (mainly of vehicular origin) were much higher than this value (~50 μg/m^3^) [23]. Unfortunately, in this country, the regulations consider a maximal daily concentration of 60 μg/m^3^ to be acceptable.

The fuel used in this study was metropolitan diesel (10 ppm sulfur with a blend of 5% soybean biodiesel).

#### Sampling and PM2.5 characterization

The 1-hour concentration of PM2.5 was determined gravimetrically using Harvard impactors (Air Diagnostics, Harrison, ME) at a flow rate of 10 L/m. The impactors were equipped with 37 mm polycarbonate filters with a 0.8-mm pore size (Isopore Membrane Filters polycarbonate, 0.8 mm, 37 mm; Millipore. The elements Sodium (Na), Magnesium (Mg), Aluminum (Al), Sulfur (S), Potassium (K), Calcium (Ca), Manganese (Mn), Iron (Fe), Cooper (Cu), Zinc (Zn), Rubidium (Rb), and Barium (Ba), Chromium (Cr), Arsenic (As), Vanadium (V), Silicon (Si), and lead (Pb) were quantified using an energy dispersive X-ray fluorescence spectrometer (EDX 700-HS, Shimadzu Corporation Analytical Instruments Division, Kyoto, Japan). This instrument uses a low-power Rh-target tube, voltage of 5 to 50 kV, and current of 1 to 1,000 μA. The characteristic X-ray radiation was detected with a Si (Li) detector. X-ray fluorescence emission spectra of the elements were collected for 400 s from a sample surface area of 10 mm under a vacuum atmosphere. The measured sample intensities were converted to element concentrations (ng∙cm^2^) through calibration with NIST 2783 (Air Particulate on Filter Media) standard reference materials (National Institute of Standards, Gaithersburg, MD, USA). Finally, carbon was used for mass balance [24,25]. Ten filters were collected, and all analyses were performed in triplicate (Table 1).

**Table 1:** Elementary composition of the particulate matter as assessed by an energy dispersive X-ray fluorescence spectrometer (EDX).

Elements (ng/cm^2^)	Mean	SD	Minimum	Maximum
Aluminum (Al)	97.2	24.0	61.1	141.0
Arsenic (As)	1.0	0.5	0.0	2.3
Barium (Ba)	1.7	0.5	1.0	2.7
Calcium (Ca)	22.4	6.7	10.9	36.3
Chromium (Cr)	11.5	31.7	1.3	171.4
Copper (Cu)	15.0	0.8	12.9	16.9
Iron (Fe)	59.4	136.8	15.4	746.6
Potassium (K)	35.7	13.3	2.4	55.4
Magnesium (Mg)	6.7	6.2	0.0	18.5
Manganese (Mn)	4.6	7.3	1.0	41.4
Sodium (Na)	170.9	22.0	130.0	217.0
Nickel (Ni)	9.7	4.4	6.6	31.2
lead (Pb)	6.9	2.6	1.5	12.9
Titanium (Ti)	5.8	1.3	3.2	8.3
Vanadium (V)	0.5	0.5	0.0	1.0
Zinc (Zn)	12.0	2.8	5.3	16.3
Rubidium (Rb)	1.1	0.2	1.0	1.7
Sulphur (S)	59.7	15.0	31.2	75.7
Silicium (Si)	65.3	12.5	43.7	94.0

### Experimental groups

Animals were divided into four different experimental groups:

SAL + FA: These animals received saline instillation and remained in the animal room and received filtered air.
PPE + FA: These animals received PPE instillation and remained in the animal room and received filtered air.
SAL + Diesel: These animals received saline instillation and were exposed to diesel exhaust particles.
PPE + Diesel: These animals received PPE instillation and were exposed to diesel exhaust particles.

Mice from each group were randomly selected to perform the following evaluations and there were two settings of exposure to pollutants. The experimental setting was performed to proceed with the respiratory mechanic evaluations, BAL and morphometric analysis. The second setting of pollutants exposure was performed to proceed the RT-PCR, ELISA and flow cytometry analysis.

### Respiratory mechanics

Animals were anesthetized with Thiopental (50 mg/kg, i.p.), tracheostomized and placed on a rodent mechanical ventilator (flexiVent, SCIREQ, Montreal, Canada) for respiratory mechanic assessment. They were ventilated with a tidal volume of 10 mL/kg and a respiratory rate of 120 cycles/minute. Using the previously described constant-phase model, airway resistance (Raw), tissue damping (Gtis), and tissue elastance (Htis) parameters were calculated [26].

### Bronchoalveolar lavage (BAL)

The lungs were washed with 1.5 mL of 0.9% saline and this content were recovered. The recovery volume was over 95% of the instilled fluid and was put into a test tube on ice. The white blood cells were quantified by total and differential counting. The BAL was centrifuged at 800 RPM for 10 min and the cell pellet was resuspended in 0.2 mL of sterile saline. The total number of viable cells was determined in a Neubauer hemocytometer counting chamber. The differential cell counts were performed in cytocentrifuge preparations of the BAL (450 RPM for 6 min) (Cytospin, Cheshire, UK) stained with Diff-Quick (Biochemical Sciences Inc., Swedesboro, NJ). A total of 300 cells (macrophages, lymphocytes, eosinophils and neutrophils) were counted in each Dif Quick-stained cytospin slide in a blinded fashion.

### Lung preparation

After the respiratory mechanic assessment, mice were euthanized by abdominal aorta exsanguination and lungs were removed and fixed at a constant pressure (20 cmH_2_O) using 10% buffered formalin infused through the trachea for 24 hours [27,28].

Lungs were embedded in paraffin and cut into 5-μm coronal sections for morphometry evaluation and were stained with H&E for lung structure analysis, Picrosirius (for collagen fibers) and Resorcin-Fucsin (for elastic fibers) [29].

#### Morphometry

For conventional morphometry, the tissue samples were stained with hematoxylin and eosin (H&E) to perform the mean linear intercept (Lm) measurements. Lm was obtained by counting the number of times that the lines of the reticulum containing 50 lines and 100 points, intercepted the alveolar walls in lung parenchyma. We performed the Lm analysis in distal areas of parenchyma (peripheral airspaces) and used the following equation: Lm = Ltotal/NI [30], where Ltotal is the sum of all grid segments, calculated by measuring each segment with a ruler (Carl Zeiss Microscopy GmbH, Jena, Germany) attached to the microscope, and NI (average number of times that the lines intersected the alveolar walls). All Lm values were expressed in micrometers (μm).

To evaluate the volume proportion of collagen and elastic fibers, the number of points hitting a specific fiber in the alveolar parenchyma were counted and compared with the number of points hitting the alveolar septa in each field [31]. Measurements were performed in 15 non-over fields at 400 magnifications in each animal.

#### Immunohistochemistry

Tissue sections were deparaffinized and hydrated. After blocking endogenous peroxidase activity, antigen retrieval was performed with a high-temperature citrate buffer (pH = 6.0). We used a rabbit polyclonal anti-caspase-3 sc-7148 (1:500, Santa Cruz Biotechnology, CA, USA). The rabbit secondary antibody in conjunction with Vectastain ABC Kit (Vector Laboratories, Burlingame, CA, USA) was used, and the sections were stained using chromogen diaminobenzidin (DAB, Sigma-Aldrich). The sections were counterstained with Harris hematoxylin (Merck, Darmstadt, Germany). For negative controls, the primary antibody was omitted from the procedure and BSA was used in the tissue samples.

Using the point-counting technique [32], we quantified the number of caspase-3 positive cells in each field divided by the number of points hitting the lung parenchyma, using the same reticulum at 400× magnification. Fifteen non-overlapping fields were analyzed and the results were expressed as cells/μm^2^.

### Cytokine analysis

Twenty-four hours after the exposure protocol, the lungs were quickly removed and stored in crushed ice in labeled tubes and then individually homogenized. To detect the cytokine levels of IL-12, INF-γ and TNF-α (M1 polarization), IL-4, IL-10 and IL-13 (M2 polarization) were determined by ELISA (OptEIA, BD PharMingen, Oxford, UK) using microplates (Costar, Cambridge, MA, USA) for each cytokine sensitized with specific monoclonal antibodies. After the samples were washed and, specific antibodies were added to the different cytokines conjugated to biotin. After solution containing streptoavidin-peroxidase, substrate and chromogen enzyme conjugate was added. The reaction was read at 450 nm using an M2 spectrophotometer (Molecules Devices, San Jose, CA, USA). Sample concentrations were calculated from the standard curves obtained with the recombinant cytokines, and the results were expressed in units of pg/ml.

### Real-time PCR

Gene expression was evaluated using Real-time Polymerase Chain Reaction (PCR) in a Rotor Gene (Qiagen, Valencia, CA, USA) thermal cycler and a Syber Green kit as a fluorescent marker (Qiagen, Valencia, CA, USA). Reactions occurred in the following steps: 95°C for 5 minutes, 40 cycles at 95°C for 5 seconds (denaturation) and 60°C for 10 seconds (annealing and extension). The PCR products were run on agarose gel to confirm fragment sizes and the specificity of the reaction. The primers were designed and used to quantify messenger RNA (mRNA) encoded by the genes described below. Analysis was performed using the software provided by the manufacturer. Briefly, an arbitrary number of copies of the genes of interest and constituents was calculated using a formula (1000000/2CT, where CT is the number of amplification cycles required to reach the threshold determined at the exponential phase of the curve) for each sample. The values are presented as the number of copies relative to the control after correcting with the constitutive GAPDH.

The primers used included Irf-5 (5’-3’ sense: AATACCCCACCACCTTTTGA; 5’-3’antisense: TTGAGATCCGGGTTTGAGAT; 60°C; NM_001311083.1); Irf-4 (5’-3’sense:CAAAGCACAGAGTCACCTGG; 5’3’antisense:TGCAAGCTCTTTGACACACA; 60°C; NM_013674.2); Cxcl-9 (5’-3’ sense: TGCACGATGCTCCTGCA; 5’-3’antisense: AGGTCTTTGAGGGATTTGTAGTGG; 60°C; NM_008599.4); Cxcl-10 (5’-3’ sense: GACGGTCCGCTGCAACTG; 5’-3’antisense: GCTTCCCTATGGCCCTCATT; 60°C; NM_021274.2); Arg1 (5’-3’ sense: GCACTCATGGAAGTACACGAGGAC5’3’antisense:CCAACCCAGTGATCTTGACTGA; 60°C; AB047402.1) and Gapdh: (5’-3’ sense: CCACCACCCTGTTGCTGTAG; 5’-3’antisense: CTTGGGCTACACTGAGGACC; 60°C; NM_008084).

### Pulmonary digestion and flow cytometry

After 24 hours at the end of the 60-day protocol, the lungs were quickly removed, washed in cold PBS, and placed in 50-ml tubes (Thermo Fisher Scientific, Inc., Foster City, USA) containing 5 ml of cold R-10 medium (RPMI supplemented with 10% fetal bovine serum), 2 mM l-glutamine, 1 mM sodium pyruvate, 1% vol/vol nonessential amino acids solution, 1% vol/vol vitamin solution (Thermo Fisher Scientific, Inc., Foster City, USA), 100 U/ml penicillin-streptomycin (Sigma-Aldrich) and 55 mM 2-ME (Thermo Fisher Scientific, Inc., Foster City, USA). The lungs were then transferred to 50-ml tubes containing 4 ml of prewarmed digestion solution R-10 medium, 0.5 mg/ml Collagenase-type IV (Thermo Fisher Scientific, Inc., Foster City, USA) and 30 μg/ml DNAse (Sigma-Aldrich). The lungs were next cut into small pieces (1 mm) with scissors and placed in a shaker at 180 RPM and 37°C for 1 hour. The digestion solution was passed through 40-μm cell strainers (BD Biosciences, MD, USA), placed in new 50-ml tubes and the remaining pieces were smashed with syringe plungers until all of the tissues were dissolved. The cell strainers were washed with 5 ml cold PBS, and the 50 ml tubes were centrifuged at 1500 RPM and 4°C for 5 min. The supernatants were discarded, the red blood cells were disrupted with 1 ml of ACK lysing buffer (Thermo Fisher Scientific, Inc., Foster City, USA) and the tubes were centrifuged at 1500 RPM at 4°C for 5 min. The supernatants were discarded and the lung cells resuspended in 500 μl of R-10 medium to proceed with cell culturing. The cell suspensions were placed in a flat bottom 96-well plate (Corning, MA, USA) in a final volume of 200 μl per well and stimulated with LPS (1 μg/ml) for 4 h. Brefeldin A (eBioscience, Thermo Fisher Scientific, Inc, Foster City, USA) was added after 1 h of stimulation. The plate was centrifuged at 1500 RPM at 4°C for 5 min, and the cells were fixed and permeabilized with cytofix/cytoperm (BD Bioscience, Thermo Fisher Scientific, Inc, Foster City, USA) for 15 min at room temperature. The cells were washed with perm/wash and stained with anti-CD45 PercP, anti-CD11b APCCy7, anti-CD11c BV421, anti-CD64 PE, anti-IL-10 APC and anti-TNF-α PECy7 (all from BD Biosciences). The samples were acquired with a FACs Canto II (BD Biosciences) and analyzed using FlowJo software (Tree Star) [33].

### Statistical analysis

Statistical analysis was performed using the SigmaStat program (version 11.0; Systat Software, San Jose, CA, USA). We performed the Shapiro-Wilk test followed by Levine test to evaluate the normal distribution and the analysis of variance, respectively.

Since all analyzed parameters have passed either on the normality or equal variance test, we used the two-way ANOVA test followed by a parametric multiple-comparison test (Holm-Sidak). Results were expressed as means ± standard error (SE), and a *p* value less than 0.05 was considered statically significant.

## Results

### Pollutants measured in real time

The PM2.5, NO and NO_2_ concentrations were in accordance with the WHO ambient air quality standards [22]. The temperature and humidity within the chamber were similar among the groups and in accordance with the standards of care for rodents (Table 2).

**Table 2:** Average exposure concentrations of pollutants (PM2.5, NO and NO_2_), temperature and relative humidity measured inside the chamber in real time for the experimental group exposed for 60 days.

Parameters	Time				
	60 days				
	Mean	SD	Minimum	-	Maximum
PM2.5 (μg/m^3^)	577	112	317	-	867
NO (μg/m^3^)	16	18	1	-	72
NO_2_ (μg/m^3^)	141	243	0	-	1120
Temperature (°C)	21	4	14	-	30
Humidity (%)	69	9	38	-	83

PM2.5, particulate matter with aerodynamic diameter less than 2.5 micrometers; NO, nitric oxide; NO_2_, dioxide nitrogen.

#### Respiratory mechanics

**Fig 1:**
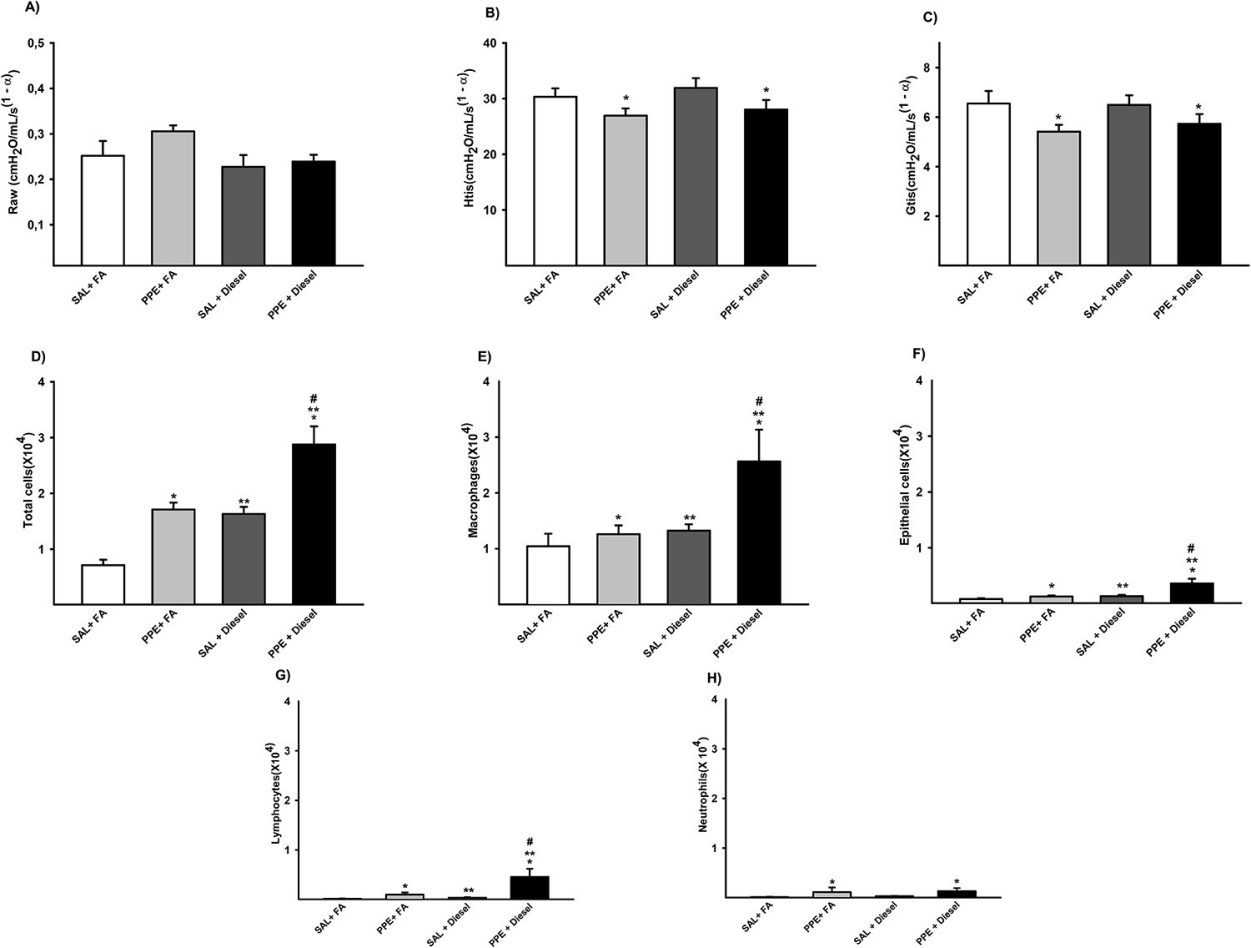
Parameters of respiratory mechanic for Raw (A), Htis (B) and Gtis (C) are expressed as the mean ± SE (SAL + FA group: n = 9, PPE + FA group: n = 9, SAL + Diesel group: n = 14 and PPE + Diesel group: n = 10). (A) No significant difference for this parameter. (B) F = 7.774; df = 1; *p = 0.008 compared with the SAL + FA and SAL + Diesel groups (C) F = 5.356; df = 1; *p = 0.026 compared with the SAL + FA and PPE + FA groups. Values of total cells (D), macrophages (E), epithelial cells (F), lymphocytes (G) and neutrophils (H) in BAL are expressed as the mean ± SE. (D) F = 33.067; df = 1; *p ≤ 0.001 compared with the SAL + FA and SAL + Diesel groups. F = 28.636; df = 1; **p ≤ 0.001 compared with the SAL + FA and PPE + FA groups. F = 0.404; df = 1; #p < 0.001 compared with the PPE + FA and SAL + Diesel groups (SAL + FA group: n = 10, PPE + FA group: n = 10, SAL + Diesel group: n = 14 and PPE + Diesel group: n = 13). (E) F = 4.363; df = 1; *p = 0.043 compared with the SAL + FA and SAL + Diesel groups. F = 5.157; df = 1; **p = 0.028 compared with the SAL + FA and PPE + FA groups. F = 2.166; df = 1; #p = 0.012 compared with the PPE + FA and SAL + Diesel groups (SAL + FA group: n = 10, PPE + FA group: n = 10, SAL + Diesel group: n = 14 and PPE + Diesel group: n = 13). (F) F = 7.414; df = 1; *p = 0.009 compared with the SAL + FA and SAL + Diesel groups. F = 8.117; df = 1; **p = 0.007 compared with the SAL + FA and PPE + FA groups. F = 3.268; df = 1; #p = 0.002 compared with the PPE + FA and SAL + Diesel groups (SAL + FA group: n = 10, PPE + FA group: n = 10, SAL + Diesel group: n = 14 and PPE + Diesel group: n = 13). (G) F = 5.884; df = 1; *p = 0.020 compared with the SAL + FA and SAL + Diesel groups. F = 4.861; df = 1; **p = 0.033 compared with the SAL + FA and PPE + FA groups. F = 3.976; df = 1; #p < 0.006 compared with the PPE + FA and SAL + Diesel groups (SAL + FA group: n = 10, PPE + FA group: n = 10, SAL + Diesel group: n = 14 and PPE + Diesel group n = 13). (H) F = 4.590; df = 1; *p = 0.039 compared with the SAL + FA and SAL + Diesel groups (SAL + FA group: n = 10, PPE + FA group: n = 9, SAL + Diesel group: n = 13 and PPE + Diesel group: n = 12).

In respiratory mechanic analysis, we observed that animals that were administered PPE (PPE + FA and PPE + Diesel groups) presented a significant decrease in Htis values and in Gtis when compared to those animals that did not receive PPE (SAL + FA and SAL + Diesel groups). No significant difference was observed in the Raw parameter (Fig 1A–1C).

### Bronchoalveolar lavage (BAL)

When we analyzed BAL, we observed that animals that were administered PPE or exposed to diesel exhaust particles presented an increase in the total number of inflammatory cells compared to those animals that received saline and were exposed to filtered air (SAL + FA group). In addition, we observed increasing values in the number of macrophages, epithelial cells and lymphocytes in these groups compared to those of the SAL + FA group. However, we only observed increased values in the number of neutrophils in the PPE groups (PPE + FA and PPE + Diesel) compared to those of the other groups.

All animals that received PPE and were exposed to diesel exhaust particles (PPE + Diesel group) presented even higher numbers of total cells in BAL, macrophages, epithelial cells, and lymphocytes. (Fig 1D–1H).

### Morphometry

**Fig 2:**
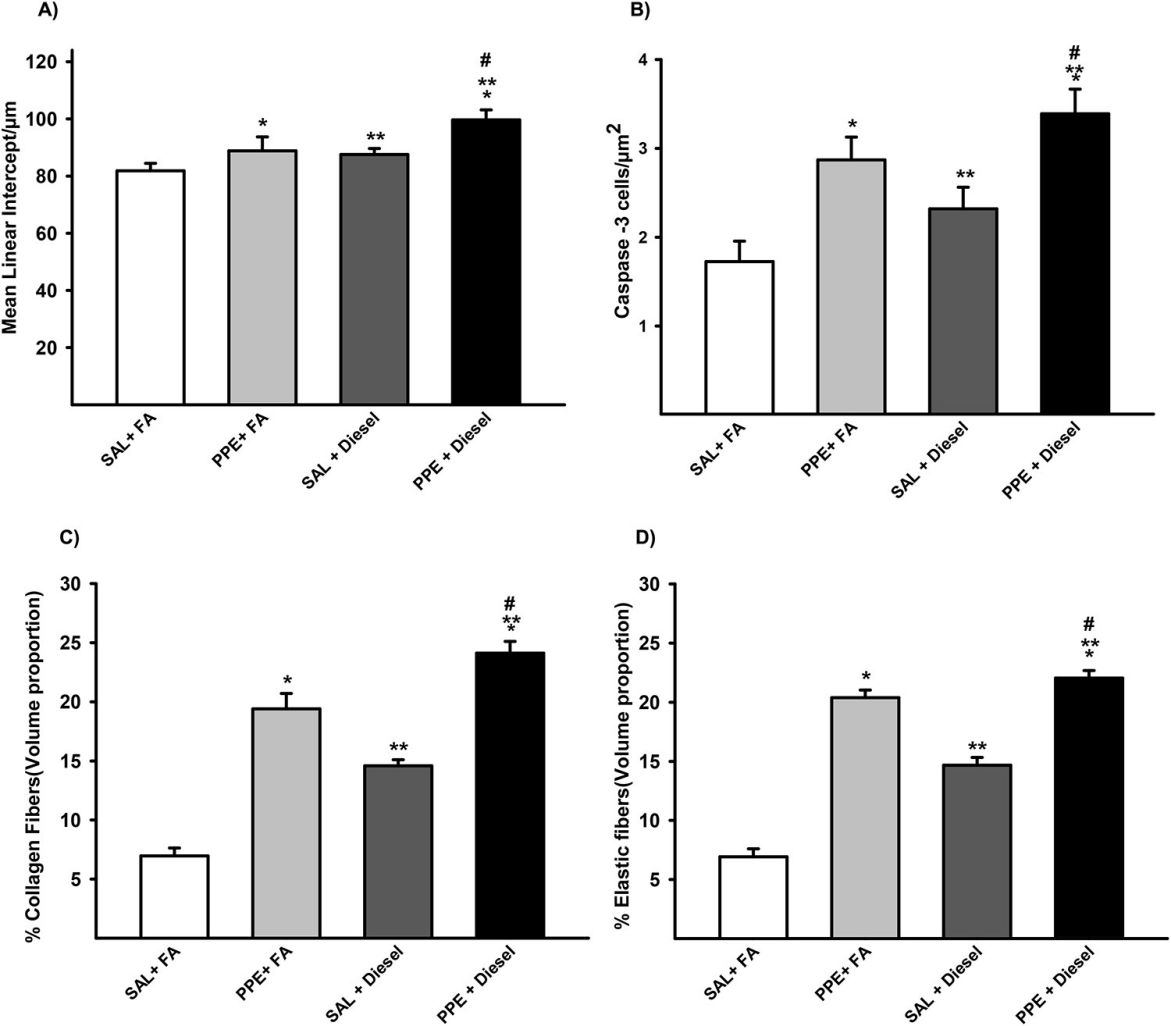
Lm (A) values in distal areas of the parenchyma are expressed as the mean ± SE (SAL + FA group: n = 7, PPE + FA group: n = 8, SAL + Diesel group: n = 11 and PPE + Diesel group: n = 10). (A) F = 21.546; df = 1; *p = 0.001 compared with the SAL + FA and SAL + Diesel groups. F = 9.702; df = 1; **p ≤ 0.004 compared with the SAL + FA and PPE + FA groups. F = 0.114; df = 1; #p = 0.023 compared with the PPE + FA and SAL + Diesel groups. The density of positive cells for Caspase-3 (B) in the lung parenchyma is expressed as the mean ± SE (SAL + FA group: n = 10, PPE + FA group: n = 9, SAL + Diesel group: n = 13 and PPE + Diesel group: n = 12). (B)F = 18.394; df = 1; *p < 0.001 compared with the SAL + FA and SAL + Diesel groups. F = 4.655; df = 1; **p = 0.039 compared with the SAL + FA and PPE + FA groups. F = 0.0213; df = 1; #p ≤ 0.005 compared with the PPE + FA and SAL + Diesel groups. Volume proportion of collagen (C) in distal areas of the parenchyma are expressed as the mean ± SE (SAL + FA group: n = 7, PPE + FA group: n = 8, SAL + Diesel group: n = 11 and PPE + Diesel group: n = 10). (C) F = 47.997; df = 1; *p < 0.001 compared with the SAL + FA and SAL + Diesel groups. F = 140.696; df = 1; **p < 0.001 compared with the SAL + FA and PPE + FA groups. F = 0.628; df = 1; #p < 0.001 compared with the PPE + FA and SAL + Diesel groups. The volume proportion of elastic fibers (D) in distal areas of the parenchyma are expressed as the mean ± SE (SAL + FA group: n = 7, PPE + FA group: n = 8, SAL + Diesel group: n = 11 and PPE + Diesel group: n = 10). (D) F = 244.562; df = 1; *p < 0.001 compared with the SAL + FA and SAL + Diesel groups. F = 49.713; df = 1; **p < 0.001 compared with the SAL + FA and PPE + FA groups. F = 21.127; df = 1; #p < 0.001 compared with the PPE + FA and SAL + Diesel groups.

**Fig 3:**
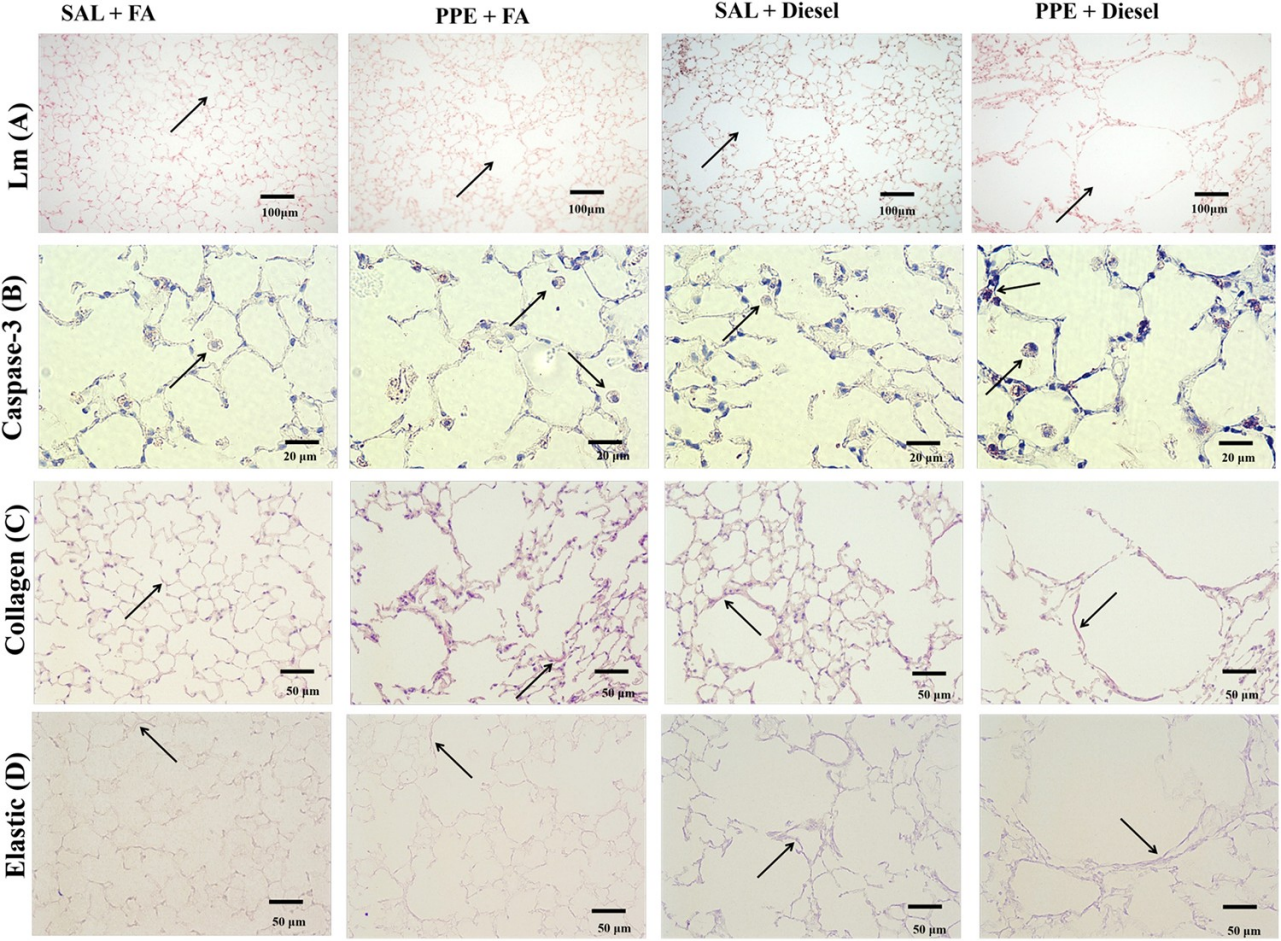
Histology photomicrographs. A representative area is shown from the pulmonary parenchyma of the experimental groups for: Mean Linear Intercept (Lm) (A), the density of positive cells for Caspase-3 (B), the volume proportion of collagen (C), and the volume proportion of elastic fibers (D).

Animals that were administered PPE or exposed to diesel exhaust particles presented an increase in Lm values compared to the animals that were only exposed to filtered air. Together, the combination of PPE instillation and diesel exhaust particle exposure induced an even greater Lm value when compared to those of the other groups (Figs 2A and 3A).

### Increased density of Caspase-3 positive cells in lung parenchyma

In immunohistochemistry analysis, we found increased Caspase-3 expression in the lung parenchyma of animals that received PPE (PPE + FA and PPE + Diesel) compared with those of the other groups and in animals that were only exposed to diesel exhaust particles (Figs 2B and 3B).

### Collagen and elastic fiber remodeling

The same pattern was observed for the percentage of collagen fibers and elastic fibers in the parenchyma. Animals that were administered PPE or exposed to diesel exhaust particles presented an increase in these fiber amounts compared with the animals that were only exposed to filtered air. The combination of PPE instillation and diesel exhaust particle exposure exacerbated this response. (Figs 2C, 2D, 3C and 3D).

### Cytokine analysis

**Fig 4:**
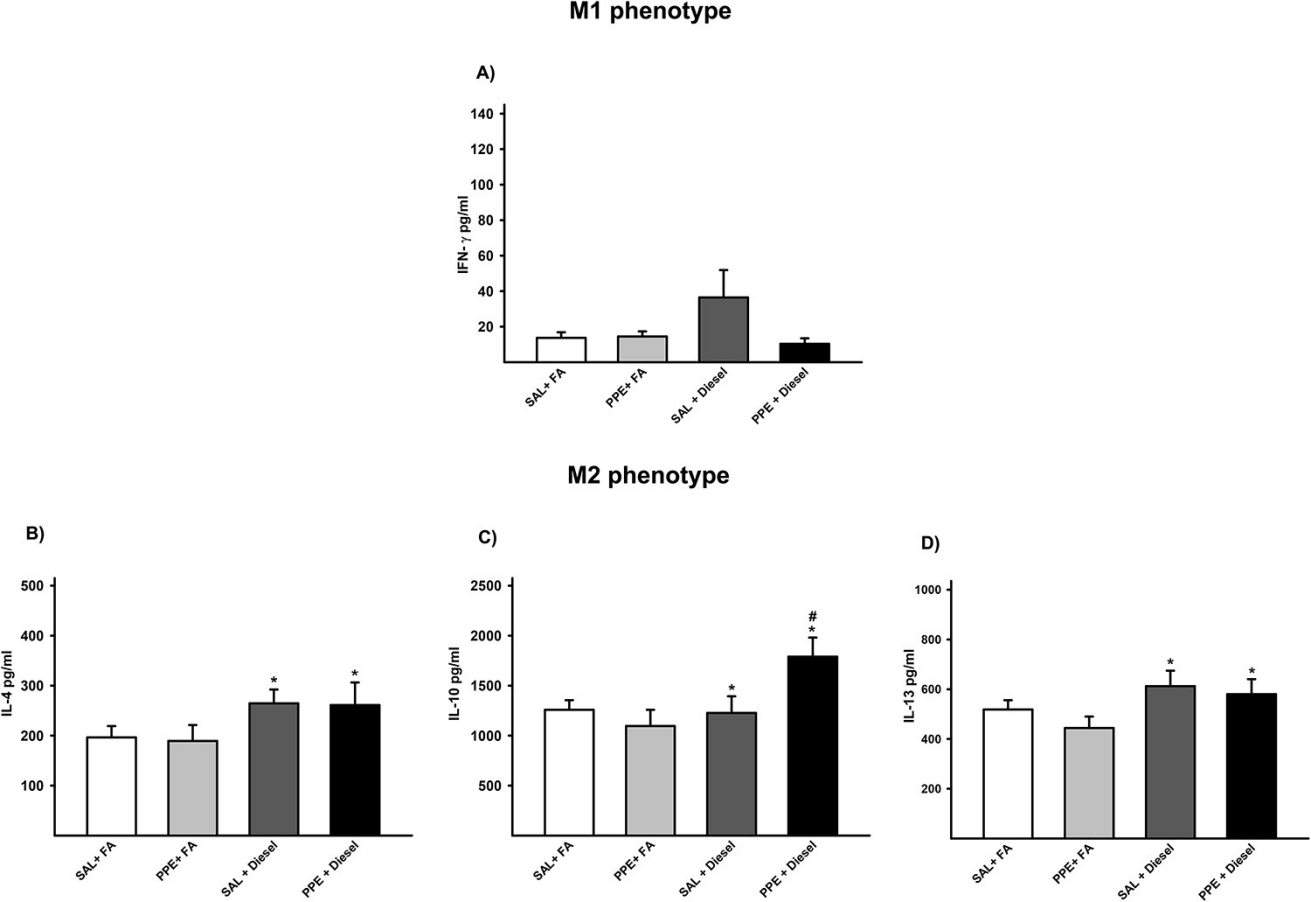
Protein quantification by ELISA for INF-γ (A), IL-4 (B), IL-10 (C) and IL-13 (D) is expressed as the mean ± SE (SAL + FA group: n = 10, PPE + FA group: n = 8, SAL + Diesel group: n = 9 and PPE + Diesel group: n = 8). (A) There was no significant difference among the experimental groups. (B) F = 4.673; df = 1; *p = 0.038 compared with the SAL + FA and PPE + FA groups (SAL + FA group: n = 10, PPE + FA group: n = 9, SAL + Diesel group: n = 9 and PPE + Diesel group: n = 9). (C) F = 1.018; df = 1; *p = 0.032 compared with the SAL + FA and PPE + FA groups. F = 4.992; df = 1; #p < 0.004 compared with the PPE + FA and SAL + Diesel groups (SAL + FA group: n = 8, PPE + FA group: n = 10, SAL + Diesel group: n = 9 and PPE + Diesel group: n = 10). (D) F = 4.787; df = 1; *p = 0.036 compared with the SAL + FA and PPE + FA groups (SAL + FA group: n = 10, PPE + FA group: n = 8, SAL + Diesel group: n = 9 and PPE + Diesel group: n = 8).

Regarding the analysis of the levels of IL-12, IFN-γ and TNF-α (M1 polarization), we did not observe differences between the groups (Fig 4A).

The groups exposed to diesel exhaust particles showed increased protein values of IL-4, IL-10 and IL-13 (M2 polarization) when compared to those of the groups that were not exposed to diesel exhaust particles. The PPE + diesel group presented even higher IL-10 values than the SAL + diesel group. These results suggest that diesel exhaust particles have an M2 polarization mechanism (Fig 4B–4D).

### Real-time PCR—Gene expression for M1 and M2 markers

**Fig 5:**
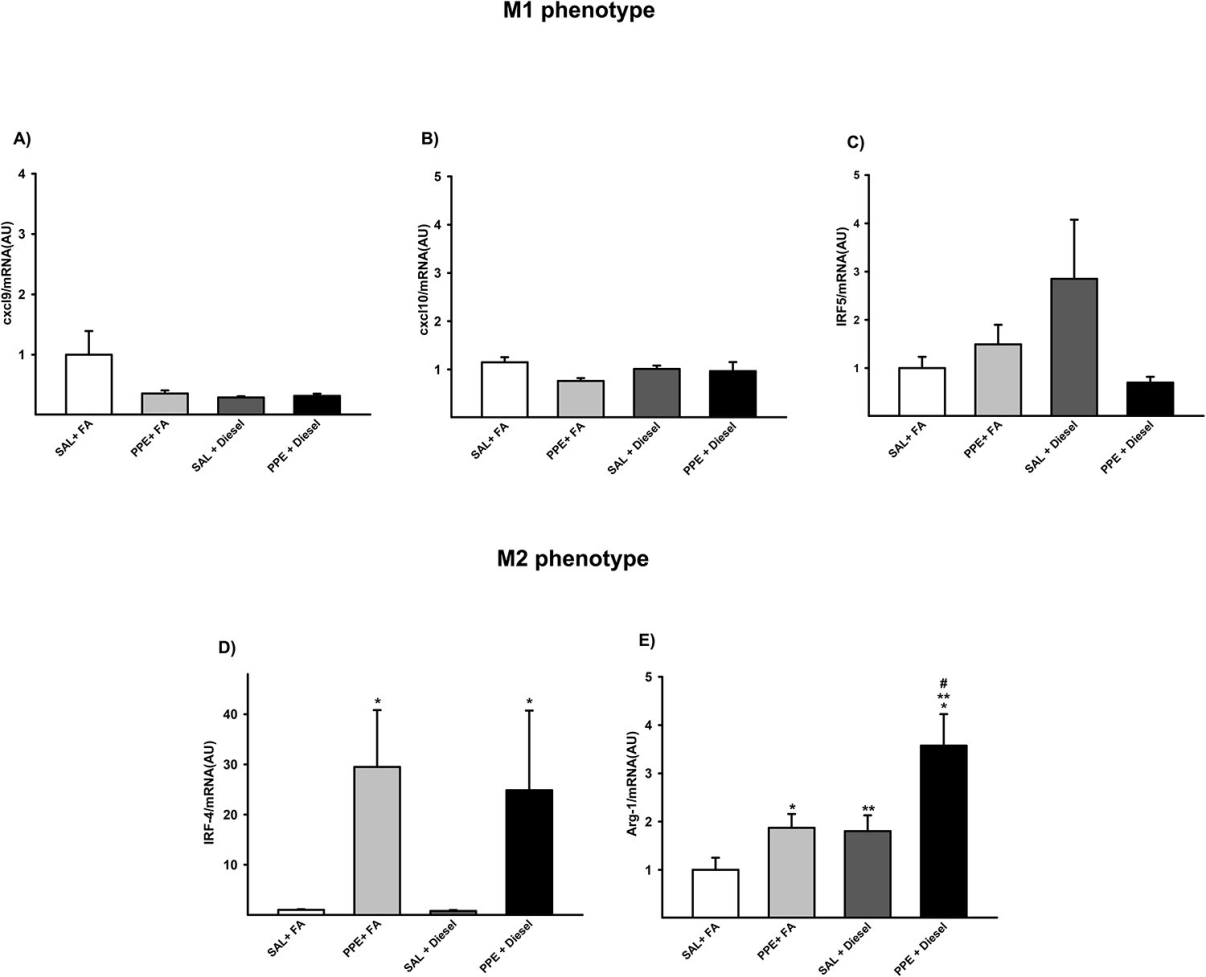
Gene expression of Cxcl9 (A), Cxcl10 (B), Irf-5 (C), Irf-4 (D), and Arg-1 (E) are expressed as the mean ± SE (SAL + FA group: n = 9, PPE + FA group: n = 6, SAL + Diesel group: n = 11 and PPE + Diesel group: n = 6). (A-C) No significant difference was observed among the experimental groups. (D) F = 5.251; df = 1; *p = 0.048 compared with the SAL + FA and SAL + Diesel groups (SAL + FA group: n = 9, PPE + FA group: n = 6, SAL + Diesel group: n = 11 and PPE + Diesel group: n = 6). (E) F = 9.348; df = 1; *p = 0.005 compared with the SAL + FA and PPE + FA groups. F = 10.401; df = 1; **p = 0.004 compared with the SAL + FA and SAL + Diesel groups. F = 1.218; df = 1; #p ≤ 0.009 compared with the PPE + FA and SAL + Diesel groups (SAL + FA group: n = 9, PPE + FA group: n = 6, SAL + Diesel group: n = 11 and PPE + Diesel group: n = 6).

No differences were detected in expression of genes associated with the M1-like phenotype (Cxcl9, Cxcl10 and Irf-5) between the groups (Fig 5A–5C).

In evaluating the gene expression of the M2-like phenotype, animals that were administered PPE (PPE + FA and PPE + Diesel) showed an increase in IRF-4 and Arg-1 expression when compared to those of the other groups. Animals exposed only to diesel exhaust particles also presented higher values of Arg-1 compared to those of the SAL + FA group. Together, PPE instillation and diesel exhaust particle exposure showed an increase value only in Arg-1 expression (Fig 5D and 5E).

### Pulmonary digestion and flow cytometry analysis of M1 and M2 polarization

**Fig 6:**
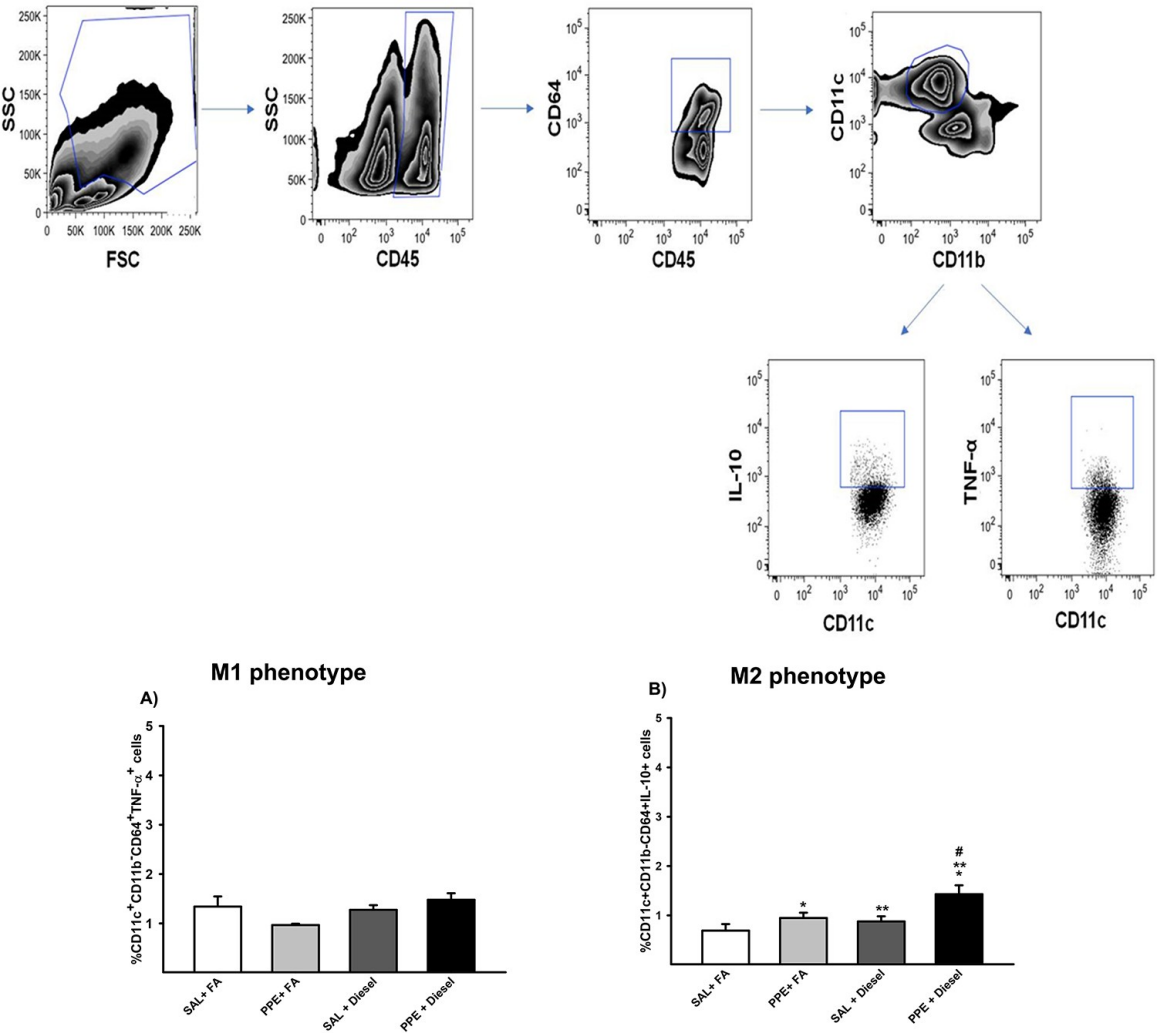
Expression of TNF-α (A) and IL-10 (B) in alveolar macrophages analyzed by flow cytometry is expressed as the mean ± SE (SAL + FA group: n = 9, PPE + FA group: n = 6, SAL + Diesel group: n = 11 and PPE + Diesel group: n = 6). No significant difference was observed among the experimental groups. (B) F = 9.056; df = 1; *p = 0.005 compared with the SAL + FA and SAL + Diesel groups. F = 6.201; df = 1; **p = 0.019 compared with the SAL + FA and PPE + FA groups. F = 1.220; df = 1; #p ≤ 0.031 compared with the PPE + FA and SAL + Diesel groups.

Regarding the analysis of M1-like phenotype polarization, no significant differences were found between the groups in terms of TNF-α analysis (Fig 6A).

Flow cytometry analysis of M2 polarization in the groups that received PPE instillation or were exposed to diesel exhausted particles revealed an increase in IL-10 in the total percentage of macrophages in the lung compared to the SAL + FA group. Together, PPE instillation and diesel exposure showed higher values of IL-10 compared to the other groups (Fig 6B).

## Discussion

Our results showed that both chronic exposure to diesel exhaust particles and PPE instillation induced an inflammatory response, leading to an enhancement in the apoptosis mechanism and increased numbers of M2-like macrophages. Such inflammatory conditions induce emphysema development and remodeling of parenchymal fibers, which were exacerbated when both factors were present.

Previous findings have shown that long-term exposure to PM10 increases the risk of COPD development and might accelerate the loss of lung function [8]. Furthermore, COPD patients have been shown to be more susceptible to the adverse effects of ambient PM [6].

Although the structural changes evaluated by Lm and volume proportion of collagen and elastic fibers showed that the combination of exposure to air pollutants and PPE instillation exacerbated emphysema development, we observed a worsening in lung function only in the PPE groups, and exposure to diesel exhaust particles did not impair this response.

The distribution of emphysema in the lung is associated with altered composition of parenchymal fibers after remodeling. Despite the increased collagen and elastic fiber amounts during the remodeling process, these newly formed fibers show a fragmented aspect and disordered distribution, which impairs the elastic properties of lung tissue [34].

The disparities between lung function and structural parameters were addressed by Anciaes et al in a temporal study. They showed that respiratory mechanics parameters did not always mirror the changes detected by histhological analysis at various time intervals after papain administration in mice. This occurred because an increase in elastic force induced by the deposition of collagen and elastic fibers may have the opposite effect on the elastic properties of pulmonary tissue promoted by a decrease in alveolar surface area [35].

Regarding the inflammatory profile, we observed an increase in lymphocytes, macrophages and epithelial cells in BAL samples, and the macrophages were predominant. Previous findings have demonstrated the importance of macrophages in the inflammatory response in PPE- and cigarette smoke-induced models [27,36–38]. Once we observed the predominance of macrophages, we proceeded to analyze the effect of microenvironmental stimuli on M1 and M2 macrophage phenotypes in lung samples.

The progression and maintenance of the inflammatory process and the increase in the prevalence of lung tissue injuries are associated with the ineffective removal of pollutant particles, especially smaller particles such as PM2.5, which reach the most distal portions of the respiratory tract (8, 11).

When remained in the pulmonary tissue, these particles are responsible for inducing the release of proinflammatory agents, intensifying and prolonging this inflammatory process and leading to cell apoptosis [8,19,39,40].

The small size of the particles emitted by diesel vehicles are composed of organic fraction components, biological materials, and transition metals that are recognized by their deleterious effects in the respiratory tract [41].

The elementary composition of our particulate matter in the exposure chamber showed high levels of metals, especially Iron (Fe). The metals contained in diesel, such as Cu, Ni and Fe, are recognized to generate oxidants and trigger an inflammatory response [42, 43], alteration in alveolar parenchyma [44] and cell apoptosis [45–48].

We showed an increase in caspase-3 positive cells, a marker of apoptotic cells, that was intensified when there was combined air pollutant exposure and PPE instillation.

These findings are in accordance with Arg-1 gene expression, as the upregulation of Arg-1 gene expression is related to an increase in the clearance of apoptotic cells and extracellular matrix components by macrophages [13,49,50].

The increased demand for phagocytosis of apoptotic cells due to exposure to pollutants likely induces an increase in Arg-1 gene expression as well as in the expression of IL-10, attesting to the presence of M2 phenotype macrophages.

Although M2 macrophages are recognized for their ability to phagocytize apoptotic cells and exogenous particles, when the concentration of these particles reaches very high levels, a decrease in phagocytic action occurs, since macrophages cannot phagocytose a volume greater than 60% of their total volume [13,49,50].

Thus, these conditions lead to apoptosis and the release of proinflammatory agents in the pulmonary tissue, intensifying and prolonging the inflammatory process [8,19,39,40, 41, 51–55].

Although there are many differences between humans and animal models regarding the pathophysiological features of emphysema, it is important to consider that we observed a similar inflammatory profile in both organisms. However, it is important to note that there is not an animal model that resembles all pathological changes observed in this human disease. Thus, knowledge of the advantages and disadvantages of each proposed model is necessary.

## Conclusions

We believe that the predominance of M2 macrophages observed in our study occurred due to the increased demand for efferocytosis induced by exposure to diesel exhaust particles as well as by PPE instillation. Indeed, the high levels of ultrafine particles that remained in the lung site play a pivotal role in the impairment of M2 macrophage activity, leading to the emphysema development and worsening symptoms.

## Supporting information

S1 File ARRIVE checklist. (PDF)

S1 Fig Stationary diesel electrical generator. Particle generator system of filtered air (FA), diesel (D) and biodiesel (BD) exhaust. PM, particulate matter; NOx, nitrogen oxide; PI, internal pressure; lpm, liter per minute.
(TIF)
