# Supplementary figures and images for: Chronic exposure to diesel particles worsened emphysema and increased M2-like phenotype macrophages in a PPE-induced model
Source: PLoS One. 2020 Jan 31;15(1):e0228393. doi: 10.1371/journal.pone.0228393 (PMC6993960; doi:10.1371/journal.pone.0228393)

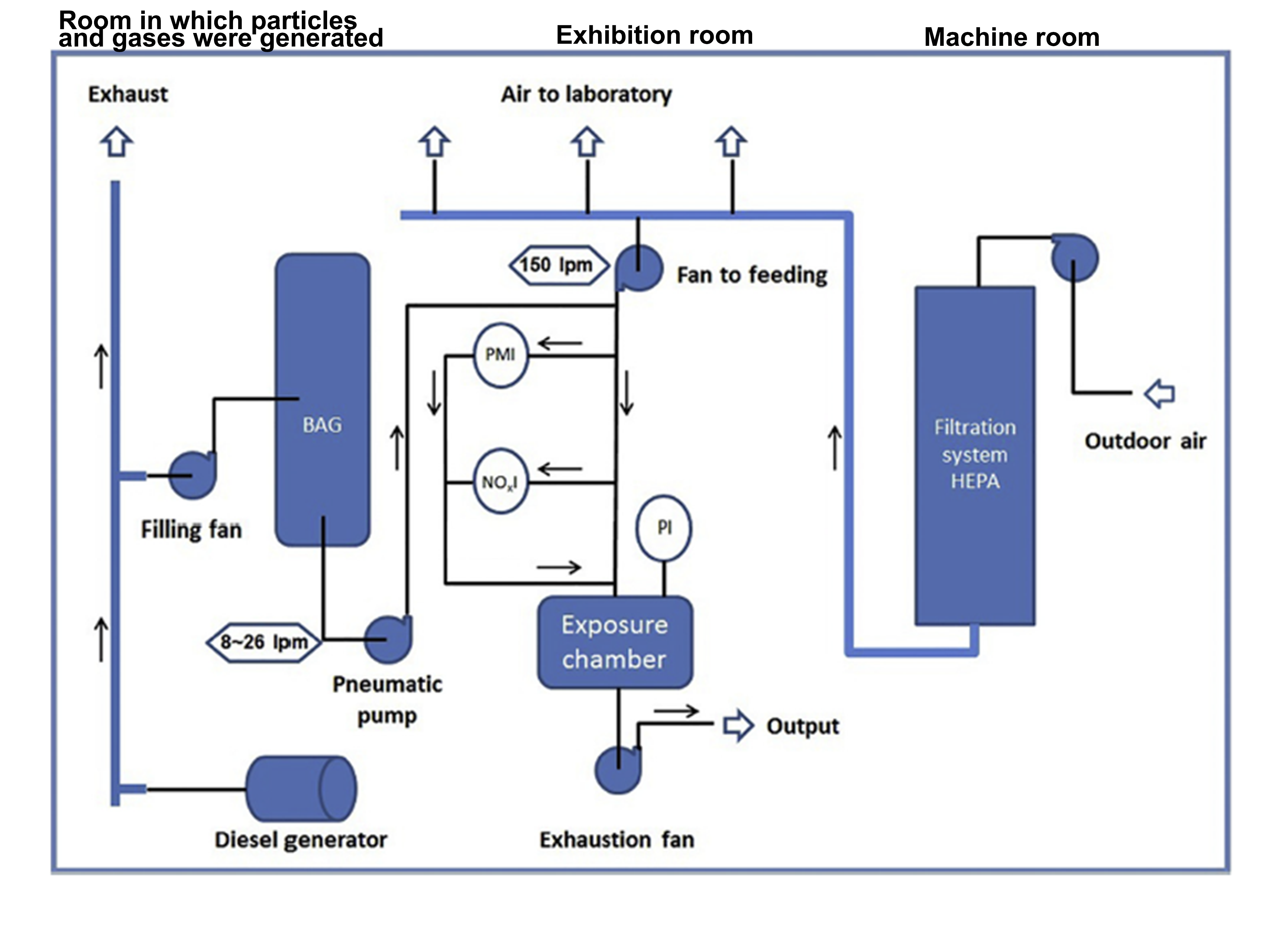

Supplement: S1 Fig — Particle generator system of filtered air (FA), diesel (D) and biodiesel (BD) exhaust. PM, particulate matter; NOx, nitrogen oxide; PI, internal pressure; lpm, liter per minute. (TIF) [file pone.0228393.s002.tif]
